# Evaluation of liver transplantation services in Kazakhstan from 2012 to 2023

**DOI:** 10.1038/s41598-024-60086-7

**Published:** 2024-04-23

**Authors:** Yuliya Semenova, Altynay Beyembetova, Saule Shaisultanova, Aruzhan Asanova, Aliya Sailybayeva, Sholpan Altynova, Yuriy Pya

**Affiliations:** 1 School of Medicine, Nazarbayev University, 010000 Astana, Kazakhstan; 2 RSE on PCV “Republican Center for Coordination of Transplantation and High-Tech Medical Services”, Ministry of Health, 010000 Astana, Kazakhstan; 3 Corporate Fund “University Medical Center”, 010000 Astana, Kazakhstan

**Keywords:** Liver transplantation, Liver fibrosis and cirrhosis, Viral hepatitis, Survival, Time series, Kazakhstan, Public health, Hepatology

## Abstract

There is a scarcity of publications evaluating the performance of the national liver transplantation (LTx) program in Kazakhstan. Spanning from 2012 to 2023, it delves into historical trends in LTx surgeries, liver transplant centers, and the national cohort of patients awaiting LTx. Survival analysis for those awaiting LTx, using life tables and Kaplan–Meier, is complemented by time series analysis projecting developments until 2030. The overall per million population (pmp) LTx rate varied from 0.35 to 3.77, predominantly favoring living donor LTx. Liver transplant center rates ranged from 0.06 to 0.40. Of 474 LTx patients, 364 on the waiting list did not receive transplantation. The 30-day and 1-year survival rates on the waiting list were 87.0% and 68.0%, respectively. Viral hepatitis and cirrhosis prevalence steadily rose from 2015 to 2023, with projections indicating a persistent trend until 2030. Absent targeted interventions, stable pmp rates of LTx and liver transplant centers may exacerbate the backlog of unoperated patients. This study sheds light on critical aspects of the LTx landscape in Kazakhstan, emphasizing the urgency of strategic interventions to alleviate the burden on patients awaiting transplantation.

## Introduction

Liver failure, marked by the liver's inability to adequately perform metabolic and synthetic functions, is a growing public health concern^1^. Acute hepatic failure progresses rapidly within 12 weeks, with drug-induced liver injury prevalent in developed nations and viral hepatitis in developing countries^2^. Chronic liver failure, lasting over 6 months, signifies progressive deterioration of liver function commonly associated with liver fibrosis and cirrhosis^3^. Despite recent advancements in managing viral hepatitis through widespread vaccination against hepatitis B and improved hepatitis C treatment, cirrhosis prevalence is increasing. It ranked as the 11th leading cause of death and 15th leading cause of morbidity in 2016^4^.

Liver transplantation (LTx) is the primary treatment for patients with acute and chronic end-stage liver failure when medical therapy is ineffective^5^. Various scoring systems exist to select suitable candidates^6,7^. Despite advancements in LTx techniques, successful surgery requires an inter-professional team and significant financial resources^8^. Two donor sources are used: living and deceased. In the Western world, deceased donor transplants exceed 90%, while in many Asian countries, living donors contribute to 24% of global LTx in 2022^9^. By 2022, the Global Observatory on Donation and Transplantation (GODT) reported 37,436 LTx surgeries, an 8% increase from 2021^10^. However, demand continues to outpace supply, leading to patients on waiting lists either succumbing to their condition or becoming unsuitable candidates for LTx^9^.

Kazakhstan, a former Soviet state in Central Asia, initiated LTx in 2012. Like in other Asian countries, living donor transplantations are more prevalent than cadaveric LTx, contributing to one of the lowest LTx rates globally^10^. However, there is a lack of comprehensive evaluations of national LTx programs, with existing reports often limited to single-center activities^11–13^. Therefore, this study aimed to evaluate the national LTx service over a 12-year period (2012–2023). It specifically seeks to analyze historical trends in living donor and deceased donor LTx surgeries and LTx centers, projecting future developments until 2030. Additionally, this study aimed to analyze a national cohort of patients awaiting LTx, examine their survival, and assess the epidemiology of common liver disorders indicating LTx. Future projections to 2030 will be made to anticipate the demand for LTx.

## Methods

### Data collection

This study comprises three distinct phases. The timeline covered by these data spans from January 1, 2015, to December 12, 2023. In the first phase, an analysis is conducted on data related to all LTx in Kazakhstan. This data is sourced from the Transplantation Coordination Center (the Center), operating under the Ministry of Health (MoH) of Kazakhstan. The Center assumes responsibility for the maintenance of the medical information system for donors and recipients registry, which comprehensively archives a waiting list data related to various organ transplantations. The registry compiles a specific subsection dedicated to LTx awaiting patients. The data also included the number of living and cadaveric liver transplants in Kazakhstan, categorized by the year of surgery, as well as the number of LTx centers by year, and the number of LTx surgeries performed in each center also by year.

The second phase involves the analysis of official statistics concerning viral hepatitis and liver fibrosis and cirrhosis, supplemented by data obtained from the population census. From the latter database, we extracted information on patients awaiting LTx but who did not receive it. The extracted data included the date of registration on the waiting list, the current status (alive vs. deceased), and the date of death (if applicable). For patients still alive at the time of data extraction, December 12, 2023, was considered the end of the follow-up period. Additional anonymized information extracted from the waiting list encompassed patient details such as age, sex, Rhesus D antigen (RhD), and blood group.

The third stage complements the findings on the number of patients awaiting LTx, with official statistics on patients with liver diseases qualifying for LTx, as outlined in the National Standard of Care on LTx^14^. The data was obtained from the electronic register of dispensary patients of the MoH. This electronic register encompasses information on all patients registered by the healthcare facilities in Kazakhstan. Extracted data included the annual number of patients presenting with selected types of viral hepatitis, liver fibrosis, and cirrhosis at outpatient healthcare facilities in Kazakhstan. Specifically, we addressed the following International Classification of Disease 10th revision (ICD-10) codes for acute and chronic viral Hepatitis: B15.0 (Hepatitis A with hepatic coma), B16.0 (Acute hepatitis B with delta-agent (coinfection) with hepatic coma), B18.0 (Chronic viral hepatitis B with delta-agent), B18.1 (Chronic viral hepatitis B without delta-agent), B18.2 (Chronic viral hepatitis C), B18.8 (Other chronic viral hepatitis), and B18.9 (Chronic viral hepatitis, unspecified). Regarding liver fibrosis and cirrhosis, the annual number of patients with the following ICD-10 codes was extracted: K74.0 (Hepatic fibrosis), K74.1 (Hepatic sclerosis), K74.2 (Hepatic fibrosis with hepatic sclerosis), K74.3 (Primary biliary cirrhosis), K74.4 (Secondary biliary cirrhosis), K74.5 (Biliary cirrhosis, unspecified), and K74.6 (Other and unspecified cirrhosis of liver) (ICD-10 Version: 2019).

National population statistics were sourced from the Bureau of National Statistics under the Agency for Strategic Planning and Reforms of Kazakhstan^15^. This dataset included the country's population numbers from January 1, 2012, to December 12, 2023, broken down by year. This information facilitated the calculation of LTx rates, number of transplant centers per million population (pmp), and prevalence of selected types of liver disease per 100,000 population.

### Statistical analysis

Various statistical analyses were undertaken to fulfill the study's objectives. This encompassed survival analysis, Kaplan–Meier analysis, Mann–Whitney U test, Pearson’s chi-squared test and time series analysis with forecast projections.

All extracted data were organized in Excel spreadsheets. The Statistical Package for Social Sciences (SPSS) for Windows (version 24.0) was used for all data analyses, with the significance level of all statistical tests set at 0.05.

#### Survival analysis

The "Survival" function in SPSS was utilized to conduct the survival analysis of patients awaiting LTx in Kazakhstan. The primary variables were the date of registration on the waiting list and the date of death or conclusion of the follow-up period (December 12, 2023). Life tables were computed to estimate the cumulative survival at specific time intervals: 0, 30, 60, 90, 180, 360, 720, 1080, 1380, 1740, 2100, 2460, 2820, 3180, and 3540 days. The number of patients entering the interval and the number of patients withdrawing during the interval were documented. Cumulative mortality rates were calculated using the following formula: 100—cumulative survival. Kaplan–Meier analysis was employed to assess the probability that patients registered on the waiting list would survive until the end of the follow-up period, as well as the mean and median survival with 95% confidence intervals (95% CI). A graph reflecting the overall survival curve during the waiting period for LTx was generated.

The data of patients who were alive on the waiting list were analyzed in comparison with the data of patients who died without receiving LTx. Before analysis, the normality of data distribution was evaluated for continuous variables by computation of the Kolmogorov–Smirnov test and graphically by generating histograms and Q-Q plots. As the data distribution differed from normal, continuous variables were presented as median (Me) with 25th and 75th percentiles. The Mann–Whitney U test was used for between-group comparisons. All categorical variables are presented as absolute numbers and percentages, and Pearson’s chi-squared test was used for between-group comparisons.

#### Time series analysis

The “Expert Modeler” function of SPSS was used to perform the time series analysis. As an initial step, annual nationwide prevalence rates of selected types of viral hepatitis (ICD-10 codes: B15.0, B16.0, B18.0, B18.1, B18.2, B18.8, and B18.9) and liver fibrosis and cirrhosis (ICD-10 codes: K74.0–74.6) were computed per 100,000 population for the period–2015–2023. Additionally, the national pmp rates of LTx and liver transplant centers were computed for the period 2012–2023. The aggregated data encompassing annual prevalence and the Pmp rates of LTx and liver transplant centers were organized in an Excel spreadsheet, indicating the reference year for the statistics. Subsequently, the best-fit epidemiological models for each type of predictive analysis were identified. Projections of the prevalence and pmp rates of LTx and liver transplant centers were made until 2030. The projections for 2025 and 2030 were reported as estimates along with their 95% CI, and the corresponding graphs were generated.

### Ethics declaration

This study was conducted in strict accordance with the principles outlined in the Helsinki Declaration. Only completely anonymized data obtained from the Transplantation Coordination Center were analyzed. Prior to the commencement of data collection, approval from the Local Commission on Bioethics of the Corporate Fund “University Medical Center” (hereafter—the Ethics Committee) was obtained. The Ethics Committee has reviewed the case and waived the need for informed consent (as documented in the Minutes of the meeting of the Ethics Committee #3 dated July 14, 2023).

## Results

**Figure 1:**
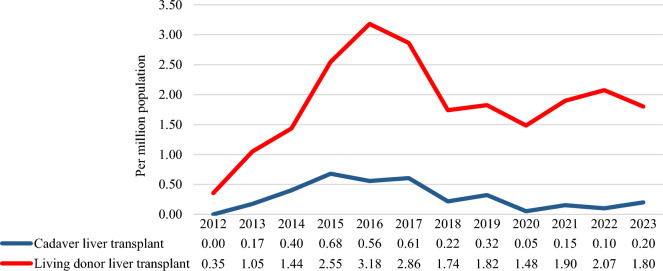
National rates of liver transplantation from living and cadaveric donors, per million population, 2012–2023.

Between 2012 and 2023, 474 LTx surgeries were performed in Kazakhstan. Among these, 411 procedures involved living donors, whereas 63 were sourced from deceased donors. No DOMINO transplantation was performed during the study period. The pmp rates of LTx were notably higher in living donors than in cadaveric transplantations. This disparity was most evident during the period—2015–2017, with pmp rates for liver donor transplants recorded as 2.55, 3.18, and 2.86, respectively. Conversely, this same timeframe witnessed an increase in cadaveric liver transplantation rates, reaching 0.68 in 2015, 0.56 in 2016, and 0.61 in 2017. The initial rise in pmp rates for liver transplants for both living and cadaveric donors declined in 2018. Subsequently, there was a resurgence in living-donor transplants in 2021, although it did not reach the rates observed during 2015–2017 (Fig. 1).

From 2012 to 2023, eight liver transplant centers operated in Kazakhstan, serving a population of approximately 20 million people, as outlined in Table 1. In 2012, when the first liver transplantation was performed in Kazakhstan, one liver transplant center conducted six surgeries. By 2013, the number of liver transplant centers had increased to five, with an average of 4.2 surgeries per center (ranging from 1 to 10). The peak number of liver transplant centers (seven in total) was reached in 2014 and 2015. During these years, the mean number of LTx per center was 4.57 in 2014 and 8.14 in 2015, with a maximum number of surgeries performed in one center reaching 8 and 13, respectively. The number of centers started to decline in 2016 and continued to decline until 2020, when there were only two liver transplant centers nationwide. However, during this same period, the maximum number of LTx procedures performed in one center peaked in 2017 (32 surgeries). Over the past three years (2021–2023), four liver transplant centers have been operational in Kazakhstan, with the mean number of surgeries per center exceeding 10.

**Table 1:** National statistics on liver transplant centers, 2012–2023.

Year	Transplant centers		Transplant surgeries	
	Number	Rate per million population	Mean number of surgeries per center	Range (min–max)
2012	1	0.06	6	N/A
2013	5	0.29	4.2	1–10
2014	7	0.40	4.57	1–8
2015	7	0.40	8.14	3–13
2016	6	0.33	11.17	4–26
2017	6	0.33	10.50	5–32
2018	5	0.27	7.20	1–22
2019	5	0.27	8.00	1–23
2020	2	0.11	14.50	4–25
2021	4	0.21	10.00	2–30
2022	4	0.20	10.75	2–26
2023	4	0.20	10.00	1–26

A total of 364 patients awaited liver transplantation since the inception of the national waiting list in 2012 and, unfortunately, did not receive liver transplantation. Among them, 181 died by the end of the follow-up period (December 12, 2023), whereas 183 remained alive. Table 2 presents comparisons between these two groups (deceased vs. alive) regarding age, sex, blood group, and RhD antigen levels, revealing no significant differences.

**Table 2:** Comparison between living and deceased patients on the national living transplant waiting list (n = 364).

Patient characteristics		Living (n = 183)		Deceased (n = 181)		*p*-value
		N	%	N	%	
Age, years (Median (25th, 75th percentile)		47.0 (38.0; 56.0)		48.0 (40.0; 55.0)		0.467*
Age group, years	< 10	3	1.6	5	2.8	0.264
	10–19	10	5.5	7	3.9	
	20–29	8	4.4	4	2.2	
	30–39	30	16.4	26	14.4	
	40–49	55	30.1	53	29.3	
	50–59	53	29.0	69	38.1	
	60–69	24	13.1	15	8.3	
	≥ 70	0	0.0	2	1.1	
Sex	Female	97	53.0	101	55.8	0.334
	Male	86	47.0	80	44.2	
Blood group	0	58	31.7	64	35.4	0.865
	A	51	27.9	50	27.6	
	B	59	32.2	52	28.7	
	AB	15	8.2	15	8.3	
Rhesus D antigen	positive	174	95.1	176	97.2	0.415
	negative	9	4.9	5	2.8	

*—test of difference was Mann–Whitney U test.

Table 3 provides insights into the survival of patients registered on the liver transplant waiting list. Of the 364 patients, 6 passed away on the day of registration on the waiting list, resulting in a cumulative survival rate of 92.0%. At the end of the first month after registration on the waiting list, 328 patients were still alive, yielding a cumulative survival rate of 87.0%. Three months after registration on the waiting list, 292 patients remained alive, with a cumulative survival rate of 83%. By the end of the first year of registration on the waiting list, only 209 patients were alive, resulting in a cumulative survival rate of 68.0%, which declined to 58.0% at the end of the second year and to 52.0% at the end of the third year. The 10-year cumulative survival rate after registration on the waiting list was 27.0%, with only seven patients alive.

**Table 3:** Life table showing cumulative survival and mortality in 364 patients registered on the national liver transplant waiting list.

Interval start time (days)	Number entering interval	Cumulative survival (%)	Number withdrawing during interval	Cumulative mortality, % (100 − survival)
0	364	92.0	6	8.0
30	328	87.0	11	13.0
60	301	85.0	3	15.0
90	292	83.0	3	17.0
180	256	78.0	1	22.0
360 (1 year)	209	68.0	5	32.0
720 (2 years)	147	58.0	1	42.0
1080 (3 years)	116	52.0	1	48.0
1380 (4 years)	93	49.0	2	51.0
1740 (5 years)	72	44.0	1	56.0
2100 (6 years)	59	41.0	0	59.0
2460 (7 years)	41	35.0	14	65.0
2820 (8 years)	18	30.0	0	70.0
3180 (9 years)	7	27.0	0	73.0
3540 (10 years)	7	27.0	0	73.0

**Figure 2:**
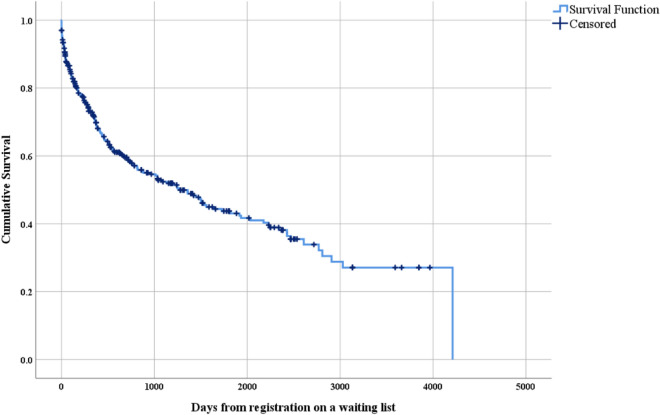
Kaplan–Meier curve displaying survival of patients awaiting liver transplantation.

Figure 2 illustrates the Kaplan–Meier survival curve of patients awaiting liver transplantation over a period of 4211 days, with a cumulative survival rate of 0.0%. The mean survival time on the waiting list was 1834.702 days (95% CI, 1615.654–2053.749), and the median survival time was 1273.0 days (95% CI, 876.611–1669.389).

To illustrate the potential need for liver transplantation, an epidemiological analysis was conducted, including the prevalence rates of selected types of liver diseases that might necessitate liver transplantation. Over the period–2015–2023, the highest prevalence rates were observed for chronic viral hepatitis B without the delta agent (ICD-10 code 18.1), increasing from 18.50 per 100,000 population in 2015 to 137.85 in 2023 (average annual increase of 28.13% (95% CI, 23.35; 33.10)). This was followed by chronic viral hepatitis C (ICD-10 code B18.2), which increased from 14.82 per 100,000 people in 2015 to 170.11 in 2023 (average annual increase of 35.29% (95% CI, 32.55; 38.08)). There was also an increase in the prevalence of liver fibrosis and sclerosis (ICD-10 codes: K74.0–74.6). The most substantial increase was observed in the rate of hepatic fibrosis with hepatic sclerosis (K74.2), rising from 0.51 per 100,000 population in 2015 to 7.72 in 2023 (average annual increase of 38.90% (95% CI, 33.06; 44.99)) (Table 4).

**Table 4:** Prevalence rates of selected types of viral hepatitis and liver fibrosis and cirrhosis, per 100,000 population.

ICD-10 codes	Year									Average change per annum, % (95% CI*)
	2015	2016	2017	2018	2019	2020	2021	2022	2023	
Viral hepatitis										
B15.0	0.02	0.03	0.03	0.04	0.04	0.05	0.05	0.05	0.05	11.34 (6.67; 16.21, *p* < 0.0001)
B16.0	0.01	0.01	0.02	0.02	0.03	0.04	0.06	0.06	0.07	30.67 (24.34; 37.32, *p* < 0.0001)
B18.0	1.39	1.95	2.96	4.26	5.97	7.48	9.31	12.41	15.89	35.33 (31.46; 39.31, *p* < 0.0001)
B18.1	18.50	24.75	36.13	51.66	68.89	79.38	91.91	112.56	137.85	28.13 (23.35; 33.10, *p* < 0.0001)
B18.2	14.82	20.33	28.90	41.58	60.01	72.42	92.07	127.13	170.11	35.29 (32.55; 38.08, *p* < 0.0001)
B18.8	0.73	0.96	1.17	1.79	2.26	2.54	2.87	3.54	4.68	25.21 (21.58; 28.96, *p* < 0.0001)
B18.9	0.36	0.46	0.68	0.98	1.40	1.64	1.77	1.75	1.76	23.75 (15.28; 32.28, *p* < 0.0001)
Liver fibrosis and cirrhosis										
K74.0	0.02	0.04	0.06	0.09	0.10	0.20	0.15	0.16	0.17	29.15 (16.48; 43.21, *p* < 0.0001)
K74.1	0.22	0.33	0.51	0.86	1.25	1.53	1.86	2.35	3.04	38.53 (31.56; 45.87, *p* < 0.0001)
K74.2	0.51	0.77	1.35	2.00	2.65	3.33	4.27	5.75	7.72	38.90 (33.06; 44.99, *p* < 0.0001)
K74.3	0.51	0.76	1.04	1.46	1.84	2.19	2.71	3.63	4.55	30.05 (26.71; 33.48, *p* < 0.0001)
K74.4	0.48	0.60	0.91	1.15	1.49	1.74	2.09	2.62	3.42	27.03 (24.01; 30.11, *p* < 0.0001)
K74.5	0.12	0.14	0.18	0.24	0.31	0.39	0.51	0.67	0.84	28.51 (27.26; 29.76, *p* < 0.0001)
K74.6	1.81	2.44	3.47	4.60	5.86	7.24	9.11	11.81	15.58	29.96 (27.86; 32.10, *p* < 0.0001)

*—95% Confidence Interval.

Table 5 presents estimates of projected prevalence rates of selected types of viral hepatitis and liver fibrosis and cirrhosis. The forecasts of liver disease are accompanied by projections of pmp rates of LTx and liver transplant centers in 2025 and 2030. According to projections, the rate of viral hepatitis with the potential to require liver transplantation will increase to 501.94 per 100,000 population in 2025 and 1081.37 in 2030, while the rate of liver fibrosis and cirrhosis will experience a more abrupt increase (54.87 per 100,000 population in 2025 and 121.04 in 2030). However, the projected pmp rates of liver transplant centers and liver transplant surgeries will remain the same in both 2025 and 2030.

**Table 5:** The projected rates of selected types of viral hepatitis, liver fibrosis and cirrhosis, liver transplantations, and liver transplant centers in Kazakhstan for the years 2025 and 2030, accompanied by 95% confidence intervals (CI).

Parameter		Year		Type of model
		2025 Rate (95% CI*)	2030 Rate (95% CI*)	
Prevalence rates, per 100,000 population	Selected types of viral hepatitis	501.94 (439.86; 564.02)	1081.37 (752.88; 1409.87)	ARIMA (0,2,0)
	Liver cirrhosis	54.87 (50.17; 59.56)	121.04 (96.18; 145.89)	ARIMA (0,2,0)
Transplant centers, surgeries, per million population	Transplant centers	0.20 (− 0.11; 0.51)	0.20 (− 0.38; 0.78)	Simple
	Transplant surgeries	2.01 (− 0.39; 4.41)	2.01 (− 2.48; 6.50)	Simple

*—95% Confidence Interval.

**Figure 3:**
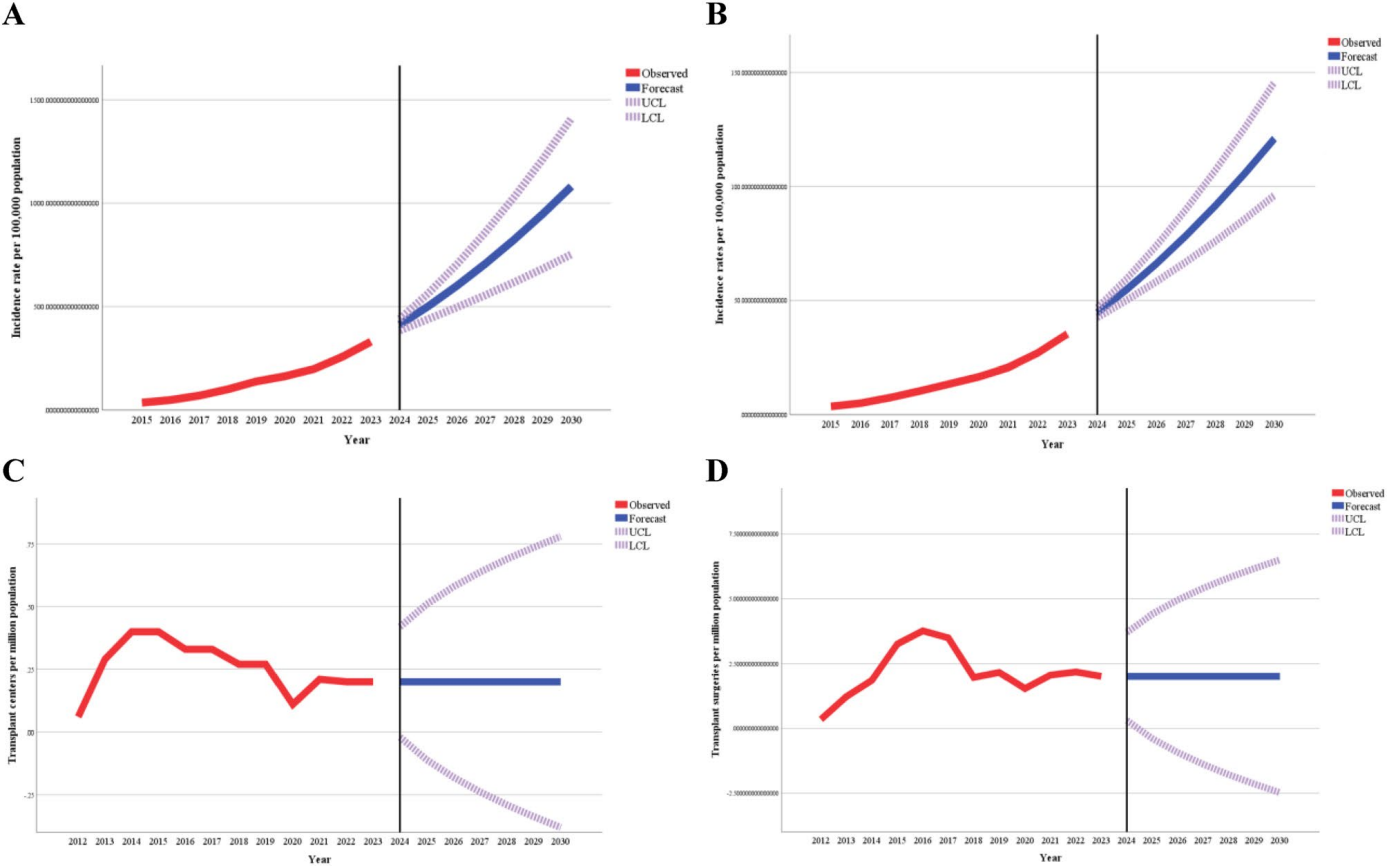
The observed and projected rates of selected types of viral hepatitis (**A**), liver cirrhosis and fibrosis (**B**), liver transplant centers (**C**), and liver transplantations (**D**) until 2030.

Figure 3 supplements the findings of Table 5, providing a graphical representation of projections until 2030. Although the forecast curves show an increase in prevalence rates for both viral hepatitis and liver fibrosis and cirrhosis, the curves remain stable for rates of liver transplant centers and transplant surgeries.

## Discussion

To the best of our knowledge, this study is the first attempt to evaluate the performance of Kazakhstan's national LTx service from its inception in 2012 to its current date in 2023. Such an evaluation is essential for improving the national LTx program to better serve the population's needs. The overall pmp rate of LTx ranged from 0.35 (2012) to 3.77 (2016), and LTx from living donors surpassed those from deceased donors multiple times. Throughout the analyzed period, the number of liver transplant centers fluctuated between one and seven for a population of approximately 20 million people, resulting in pmp rates ranging from 0.06 0.40. A total of 474 patients underwent LTx, while 364 patients were on the waiting list but did not receive transplantation. Among these, 181 patients died and 183 were still alive by the end of 2023, with no significant differences observed between these two groups. The 30-day cumulative survival rate on the waiting list was 87.0%, and the 1-year survival rate was 68.0%. The prevalence of selected types of viral hepatitis and liver cirrhosis has steadily increased from 2015 to 2023, and projections suggest that this trend will persist until 2030. Without targeted interventions, the pmp rates of LTx and liver transplant centers are expected to remain stable, contributing to the backlog of unoperated patients awaiting transplantation. These findings warrant further in-depth investigation.

According to the GODT data, in 2022, Kazakhstan ranked 11th in the list of countries based on the pmp rates of LTx from living donors, dropping from its 8th position in 2021. However, the overall pmp rates of LTx in Kazakhstan are relatively low, with Kazakhstan ranking 48th out of 91 countries in 2021 and 51st in 2022. Nonetheless, Kazakhstan stands out as the leader in LTx activities in Central Asia, surpassing other countries in the region in terms of pmp rates. Compared with other post-Soviet countries, Kazakhstan ranked 5th in 2022 and 2021, following Lithuania, Belarus, Estonia, and Georgia. In general, the pattern of LTx activities in Kazakhstan mirrors that seen in the Southeast region, characterized by a significant predominance of LTx from living donors and relatively lower overall pmp rates^10^.

Regarding transplant centers, the Pmp rates observed in Kazakhstan were lower than those in other global regions. For instance, in 2022, the region of the Americas had 2.4 liver transplant centers pmp, Europe had 3.0, the South-East region had 6.7, the Western Pacific region had 7.7, and the Eastern Mediterranean region exhibited the highest pmp rates of liver transplant centers at 13.2^10^. The number of liver transplant centers in Kazakhstan is comparable to that in the United Kingdom, where there are seven centers with a population of approximately 64 million people^16^. When considering the number of LTx surgeries performed in Kazakhstan, liver transplant centers appear to be low-volume, with the majority conducting fewer than 10 LTx per year. The only high-volume center in the country, the National Scientific Center of Surgery named after Syzganov, has performed a maximum of 32 LTx per year.

Presently, LTx services in the country are provided free of charge to residents and are funded by the health insurance fund. This funding encompasses the surgical costs for both the recipient and donor. However, ancillary expenses such as pre-surgical examinations, tests, and post-surgical rehabilitation often incur out-of-pocket expenses^17^. There are no imposed budget constraints on the number of LTx procedures conducted in the country. Nevertheless, the relatively low rates of LTx can be attributed to the opt-in approach adopted by Kazakhstan^18^. Under this approach, consent for organ donation after death must be obtained, typically granted by the deceased's relatives in the absence of declared will^19^. Generally, the opt-in approach tends to yield fewer organ transplantations than the opt-out approach, in which all deceased individuals are automatically considered potential donors. Experiences in the European region demonstrate that countries adopting the opt-out approach tend to have higher LTx rates^20^. Notably, Kazakhstan used an opt-out system, leading to more organ transplants between 2015 and 2017. However, this changed after an incident in 2017–2018 when some transplant surgeons were accused of mishandling organ transplants. Even though they were eventually cleared of all charges^21^, this incident had a lasting impact, and the pmp rates did not reach the levels of 2015–2017 even after six years.

The Transplantation Coordination Center functions as a non-profit organization, serving as a national intermediary among 40 donor hospitals and four transplant centers. This center consolidates the information on patients eligible for LTx into a unified national waiting list. The criteria for notification from donor hospitals to the Transplantation Coordination Center involve potential donors meeting the criterion of brain death. Currently, donation after circulatory death is not practiced. Throughout the study period, there was no cross-border exchange of donors or recipients with other countries in the region, and all liver transplants were performed exclusively on Kazakhstani citizens. The indication for liver transplantation is determined by a Child-Turcotte-Pugh score of 7 or higher (classes B and C)^6^, with the Model for End-Stage Liver Disease (MELD) not currently in use. Survival analysis revealed a notable proportion of patients facing mortality shortly after entering the waiting list, indicating a 13% cumulative one-month mortality. This underscores the potential benefits of earlier inclusion of these patients in the waiting list.

Over the past decade, there has been a shift in indications for LTx. End-stage liver cirrhosis remains a major indication^22^, aligned with the national standards for LTx in Kazakhstan^14^. However, the global etiology of liver cirrhosis has changed, moving away from viral hepatitis owing to the availability of effective antiviral medications and shifts in lifestyle and dietary approaches. In Kazakhstan, the burden of viral hepatitis is substantial and continues to grow, as indicated by our findings and earlier scientific data^23^. Direct-acting antiviral hepatitis C drugs have not gained widespread use in Kazakhstan, and despite antiviral hepatitis B vaccination being included in the national vaccination schedule in 1998, the prevalence of anti‐HBcore antibodies was reported to be 17.2%, surpassing rates in many other countries^24^. Projections of viral hepatitis and liver cirrhosis until 2030 indicate a likely increase in the prevalence of these diseases, suggesting a rising demand for LTx. However, it has to be acknowledged that the trend towards an increase in the rate of viral hepatitis B may be associated with an increased efficiency in reporting, rather than an absolute increase, owing to the nationwide efforts towards its prevention by means of established vaccination program.

Although liver transplantation indications among adult patients are typically preventable, such as those related to alcohol intake, viral hepatitis, and fatty liver disease, indications among pediatric patients often arise from conditions such as biliary atresia, inborn errors of metabolism, and tumors^25^. A pediatric end-stage liver disease score (PELD) is a numerical scoring system utilized to prioritize pediatric patients awaiting LTx. It is designed to assess the severity of liver disease and the risk of mortality in children under 12 years old. The PELD score considers a range of clinical parameters and designates a score from 6 to 40, with higher scores indicating a greater severity of liver disease and a higher risk of mortality. The PELD scoring system ensures that donor organs are allocated to those patients with the greatest medical need^26^.

Efforts to address the unique challenges and requirements of pediatric LTx include the establishment of specialized pediatric LTx centers. These centers offer specialized expertise in managing pediatric liver conditions, encompassing surgical techniques, perioperative care, and long-term follow-up. The introduction of a dedicated national policy for pediatric LTx could further improve the survival of children with end-stage liver disease^27^. While such provisions are not currently in place in Kazakhstan, their implementation could spearhead progress in child survival. Globally, despite advancements in pediatric LTx, significant challenges remain as many cases of pediatric liver failure present complex issues, and transplantation is only one aspect of their treatment strategy. Therefore, it is essential to continue research and develop comprehensive approaches to address the multifaceted needs of pediatric patients with end-stage liver disease^28^.

Public health action is imperative to augment LTx activities in Kazakhstan to meet the growing demand for LTx surgery. In efforts to expand the pool of available grafts, the challenging issue is described by the majority of marginal donors. These cadaver donors are associated with a higher risk of primary nonfunction and early graft impairment, leading to suboptimal long-term outcomes when compared to grafts from donors with less challenging criteria^29^. As a result, an imperative arises for active and pervasive campaigns aimed at fostering awareness regarding general population on organ donation, with the help of primary health care, mass media, community-based interventions and research institutions^30–33^.

This study had several limitations. The primary limitation is that the data available on the national waiting list of patients awaiting LTx are limited, with many specific details related to missing underlying diagnoses. This limitation makes the calculation of cause-specific survival impossible and restricts the analysis of the associated risk factors. Another limitation arises from the fact that forecast modeling utilized the prevalence rates of selected types of viral hepatitis, liver fibrosis, and cirrhosis, but data on end-stage liver disease were not available, as the study relied solely on ICD-10 codes, thereby limiting the capacity of projections. Additionally, the projected rates of both liver disease prevalence and LTx rates should be interpreted with caution, as they illustrate the need for public health action to address the existing situation. Nonetheless, this study has several strengths, the most notable being its status as the first nationwide study to analyze the outputs and outcomes of LTx services.

## Data Availability

The data utilized and examined in this study can be obtained from the corresponding author upon a reasonable request.

## Abbreviations

GODT: Global Observatory on Donation and Transplantation
ICD-10: International Classification of Disease 10th revision
LTx: Liver transplantation
MELD: Model for End-Stage Liver Disease
MoH: Ministry of Health
pmp: Per million population
RhD: Rhesus D antigen
SPSS: Statistical Package for Social Sciences
WHO: World Health Organization
